# Memory in Elementary School Children Is Improved by an Unrelated Novel Experience

**DOI:** 10.1371/journal.pone.0066875

**Published:** 2013-06-19

**Authors:** Fabricio Ballarini, María Cecilia Martínez, Magdalena Díaz Perez, Diego Moncada, Haydée Viola

**Affiliations:** 1 Instituto de Biología Celular y Neurociencias Consejo Nacional de Investigaciones Científicas y Técnicas de Argentina, Facultad de Medicina, Universidad de Buenos Aires, Buenos Aires, Argentina; 2 Departamento de Fisiología, Biología Molecular y Celular, Facultad de Ciencias Exactas y Naturales, Universidad de Buenos Aires, Buenos Aires, Argentina; University of Queensland, Australia

## Abstract

Education is the most traditional means with formative effect on the human mind, learning and memory being its fundamental support. For this reason, it is essential to find different strategies to improve the studentś performance. Based on previous work, we hypothesized that a novel experience could exert an enhancing effect on learning and memory within the school environment. Here we show that novel experience improved the memory of literary or graphical activities when it is close to these learning sessions. We found memory improvements in groups of students who had experienced a novel science lesson 1 hour before or after the reading of a story, but not when these events were 4 hours apart. Such promoting effect on long-term memory (LTM) was also reproduced with another type of novelty (a music lesson) and also after another type of learning task (a visual memory). Interestingly, when the lesson was familiar, it failed to enhance the memory of the other task. Our results show that educationally relevant novel events experienced during normal school hours can improve LTM for tasks/activities learned during regular school lessons. This effect is restricted to a critical time window around learning and is particularly dependent on the novel nature of the associated experience. These findings provide a tool that could be easily transferred to the classroom by the incorporation of educationally novel events in the school schedule as an extrinsic adjuvant of other information acquired some time before or after it. This approach could be a helpful tool for the consolidation of certain types of topics that generally demand a great effort from the children.

## Introduction

Finding different teaching strategies to improve students' performance is a subject of great relevance to education. Although arousing students' interest in the syllabus might work, this is not always possible. In particular, some topics are resisted by many students. Therefore, finding behavioral procedures with enhancing effects on human memory poses an exciting challenge, with potential implications for educational strategies.

A prevailing view in neuroscience is that memory formation is a gradual process. Memories are initially labile but they become more stable with the passage of time. This memory consolidation hypothesis was proposed more than 100 years ago [1] and some of its underlying molecular and cellular mechanisms were well characterized over the past decades [2], [3], [4]. Behavioral, hormonal and neural influences acting during this labile period can regulate memory consolidation, improving or impairing it. Thus, stress, arousal, motivation and reward can profoundly affect memory formation [5], [6], [7], [8], [9]. Moreover, there is increasing evidence supporting the view that the ability to consolidate a memory for a certain event can be concurrently improved by what has happened before or after it [10]. In this regard, previous work in rodents has demonstrated that a novel behavioral experience can promote the formation of a long-lasting memory for another task when novelty occurs within a restricted time window around the learning task [11], [12], [13], [14], [15]. This phenomenon is called *behavioral tagging* and its underlying mechanisms are currently being studied [16]. In the present work, we investigated whether a similar approach could be applied to the school syllabus during students' regular classes, with the aim of improving teaching strategies used in the educational system.

## Materials and Methods

### Students

1676 Participants (ages 7 to 9 years-old) from 8 different schools in Buenos Aires, Argentina were tested. Characteristics of the institutions and the students intervening in the experiments are shown in table S1. All students were naïve to the procedures.

### Ethics statement

This study was performed under the approval of the Ministerio de Education de la Provincia de Buenos Aires, Argentina. Also, procedures were reviewed and approved by the Head of each participating educational institution. Students were able to withdraw from the study at any time without consequence. No identifying information was collected during the study.

### General protocol

A literary memory study was carried out. Briefly, a teacher read a short story (A) to her group (X) of students (Control). In parallel, another group of students (Y) belonging to the same Institution and of the same grade was read by their own teacher another short story (B), which was associated to a novel lesson (Novelty). 24 hours after the reading of the story, long-term memory was evaluated anonymously in both groups. Two weeks later, these two groups of students were involved in a second experiment, being group X assigned the story B and group Y the story A. The stories A and B were randomly associated to control/novelty groups between different experiment in order to avoid any bias (e.g. if there were 4 different X groups, 2 of them started hearing to story A and the other one to story B as control situation).

Subjects experienced the novel lesson 1 or 4 h before or after the reading of the story. This protocol was performed with children that attend school from 7:30 am to 12:00 pm (morning shift) or 13:00 pm to17:30 pm (afternoon shift).

Long-term memory was evaluated using a written list of 10 questions of different difficulty levels -easy, intermediate and hard- related to the story read the day before. The answers to the three easy questions were used to check that the student paid attention and understood the reading. One point per each correct answer was assigned, and **Memory index** was calculated as the ratio between the score obtained for both the hard and the intermediate questions for students belonging to the Novelty group relative to the mean obtained for its corresponding Control group. Both short stories were appropriate for the instruction level of the students and had a comparable complexity (this assumption was based on the number of correct answers from Control groups).

The other parameter analyzed in this study is **the percentage of** **correct answers** classified by levels of difficulty. This was calculated as follows: first, it was we obtained the proportion of correct answers for each separate category –easy, intermediate and hard- for each group of students. Afterwards, a mean of the proportions from all the courses was obtained for each category. These means were used to compare the scores between Control and Novelty groups (i.e. to analyze the effect of novelty in each category) in the intermediate and hard questions. Easy questions were taken as a positive control of the students' attendance to the lesson and their comprehension of the story. To illustrate the procedure, here we include an example: to calculate the number of correct answers from a group of 30 students in control or novelty conditions, there were 90 possible answers (three questions per each student, corresponding to one level of difficulty) in each condition. If control group correctly answered 18 questions out of 90, then their score is 20%. If the novelty group correctly answered 54 out of those 90 questions, their score is 60%.

Easy questions were based on major aspects of the story such as characteŕs name and core events in the plot. These questions were correctly answered by 80% of the students in the control groups (Figures S1, S2, S3). Intermediate difficulty questions addressed relevant aspects of the story, although only mentioned once. These questions were correctly answered by about 40% of the students in the control groups (Figures S1, S2, S3). Hard difficulty questions were made on details and events unrelated to the core of the plot. These questions were correctly answered by 20% of the students in the control groups (Figures S1, S2, S3).

### Specific time-task protocol

Using a similar approach as described before, students were read two stories (A and B) on the same day, with an interval of three hours between sessions. An hour after the second short story had been read, a novel science class was presented. 24 h later, LTM for one of the stories was evaluated on one half of the students while the other story was evaluated on the second half. As a control parallel groups of students were read two different stories (randomized alternate) separated by a 3 hours interval. 24 h later, LTM was tested for both stories as described above.

### Novel lessons

In this work we considered it to be a novel activity if the class/lesson (science or music) complied with these requirements:

1. The whole group of students was unexpectedly taken from its classroom and led to a different place to attend a lesson that was not previously informed about until it started.
2. This novel lesson was given in a place inside the school but not usually frequented by the students for their lessons, e.g., common room, laboratory, patio, hall.
3. The lesson was given by a skilled teacher unknown to the students.
4. It is a short activity (20 min) never experienced before by the students, with novel contents appropriate for their age.
5. Students are encouraged to actively participate and be attentive at all times. When the activity finishes, they return to their habitual classroom.

The novel science class was based on simple experiments aiming to the constant participation of the students and their full interaction with the elements. In the first years at school it is extremely infrequent to experience this kind of empirical science lessons. In the class, students were introduced to some basic physics and biology principles such as density, gravity, superficial tension and electrostatics) with playful activities. The novel science lesson was specially designed and given by the researchers. Besides, the novel music class was planned and dictated by the music therapist aiming to resemble those novel features of the science class. Likewise, students were not informed of this class until its start and were involved in its activities. Considering that students habitually have music lessons in their schools (as it is a compulsory subject), the novel music lesson was based on innovative teaching methods and it also dealt with topics highly infrequent for the students. The lesson consisted of games with homemade instruments (like rods, x-ray films, buckets and balls) and it was intended to show that music could even be made with simple elements and yet they could also form an orchestra with those atypical instruments.

### Familiar lesson

The same music lesson described above was presented for 2 weeks (one lesson each week) and in the third week this “familiar lesson” was associated with a short story read 1 hour before the music lesson. The following day, long-term memory for the story was tested.

### Complex figure test

We designed a task to test graphic memory based on Rey-Osterrieth's complex figure test [17]. A complex figure picture (Figure S4) was shown to the whole group of students and each student was allowed to copy it on a blank paper for a 2-minute period. Once finished, drawings were handed in to the teacher. 24 hours later, the memory of this figure was tested by asking students to draw it again on a blank paper in a 4-minute interval. **Memory percentage** for each student was assessed by comparison of the copy (acquisition session) with the second drawing (test session). Each of the elements in the figure were analyzed for location, accuracy and organization by using the Rey-Osterrieth's score [18] (Table S2) and an individual score was obtained. Only those students who had completed more than 75% of the copy were included in the experiment.

Another parameter analyzed in this experiment is the **correct performance percentage** classified by levels of visual complexity. Configural elements are considered to be the most “global” parts of the design, the details as the most “local” elements (Picture S1). An average was obtained from the individual performances for both configural elements and for details. Literary and graphical activities were performed by each group's own teacher inside the classroom as part of a regular class.

For more experimental details, see Text S1.

## Results

**Figure 1:**
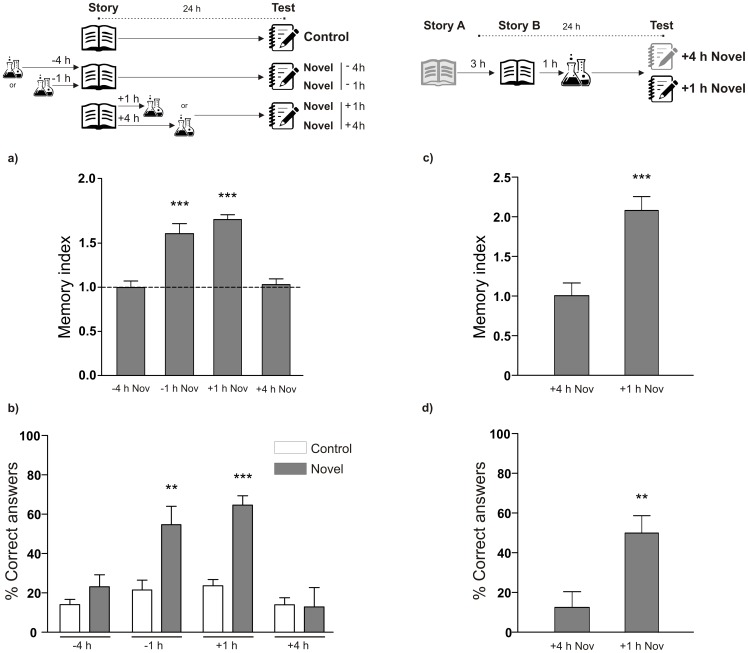
Memory of a story is enhanced by a novel science lesson around the time of the reading. A) Novelty was experienced before or after the reading (−4 h, n = 57; −1 h, n = 56; +1 h, n = 119; +4 h, n = 98). Schematic representation of the experimental protocol is presented on the top left of the panels. Memory index is shown as mean ± SEM of the ratio between control (dotted line, n = 89) and novelty groups. *** p<0.001 vs. Control, −4 h and +4 h, Newman-Keuls after one-way ANOVA. B) Mean ± SEM of the percentage of correct answers to the hard level of difficulty questions. Novel groups (−4 h, n = 6; −1 h, n = 9; +1 h, n = 9; +4 h, n = 9, calculated from students analyzed in A) vs. their controls for each time point. ** p<0.01, ***p<0.001 vs. Control, Student's t test. C) A story A was read and 3 hours later story B was read. Novelty was experienced one hour after story B. Schematic representation of the experimental protocol is presented on the top right of the panels. Memory index is shown as mean ± SEM for the novelty groups at different times. Half of the students were tested on story A (n = 20) and the others on story B (n = 28). *** p<0.001 vs +4 h, Student's t test. D) Means ± SEM of the percentage of correct answers to hard level of difficulty questions are shown corresponding to groups of students analyzed in C (+4 h, n = 6; +1 h, n = 6). ** p<0.01 +1 h vs. +4 h, Student's t test.

Using cognitive memory tests we evaluated the consequences of undergoing a novel experience in the school environment, before or after a standard learning session. In our first experiment, this session consisted of a short story that was read to elementary school students, between ages 7 and 9 (Table S1). Twenty-four hours later we evaluated how much they remembered about it with a questionnaire. The novel experience was science lesson with contents completely unknown by the students (See Text S1). Memory improvements were observed in those groups of students who had experienced the novel science lesson 1 hour before or after the story telling, but not 4 hours before or after it (Fig. 1A). The test list included questions of different levels of difficulty –easy, intermediate and hard- (See Text S1). The percentage of correct answers to hard questions was increased in those groups of students who had a novel science lesson 1 hour before or after the reading of the story, but not 4 hours before or after it (Fig. 1B). A similar outcome was observed in the case of intermediate difficulty answers, without any significant changes in the easy ones (Fig. S1).

**Figure 2:**
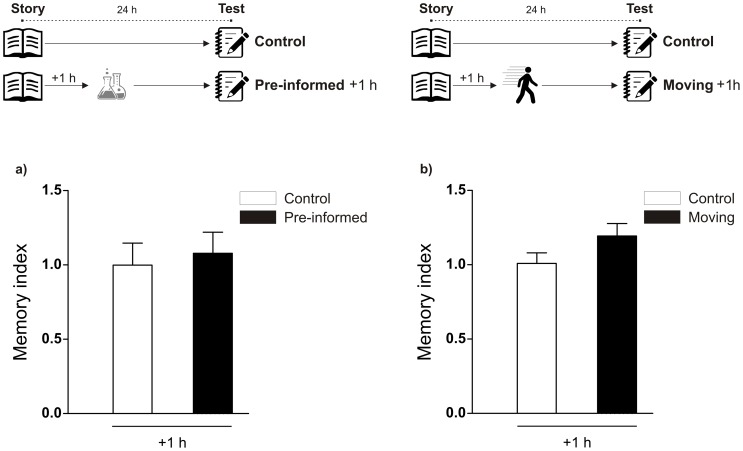
Memory of the story is not enhanced when the students were pre-informed about the contests of a new science lesson or when just moved around the classroom. Schematic representation of the experimental protocol is presented on the top panels. A) Memory index for the story is shown as mean ± SEM for the Control group (n = 25) and Pre-informed group (n = 22). A short story was read to a group of students that had been previously informed that they would be given a science class outside their habitual classroom one hour after the reading. LTM of the story was evaluated 24 hours after. p>0.05 Students t test. B) Memory index for the story is shown as mean ± SEM for the Control group (n = 25) and Moving group (n = 30). A short story was read and one hour after students moved from the classroom to the place where the novel class would take place and back, without experiencing any class. LTM of the story was evaluated 24 hours after p>0.05 Students t test.

In addition, to assess the tasks' time-specificity of this effect, students were read two different stories separated by a 3-hour interval, being only one of them followed by a novel lesson 1 hour later. Memory enhancement was observed for the story in the adjacent context of novelty but not for the other, in accordance to the time-dependence effect of novelty observed in Fig. 1A (Fig. 1C). As a control, parallel groups of students were read two different stories separated by a 3 hour interval and LTM was tested for both stories. When the stories were not associated with a novel lesson, there was no difference between the scores obtained for each of them. (Memory index for Story A = 1.001±0.09470, n = 39 vs Story B = 1.080±0.1050, n = 47; p>0.05 Student's t test). Moreover, the percentages of correct answers to the hard (Fig. 1D) and intermediate difficulty questions (Fig. S2) were significantly increased for the story read close to novelty in relation to the story read 4 hours before it. Easy questions were correctly answered by a similar percentage of students in both groups (Fig. S2). This suggests the effect of novelty accounts for the enhancement of memory on the harder tasks. In addition, it is important to note that the promoting effects of novelty lessons were not merely due to moving around. The control group of students that only moved from the classroom to the place where the novel class took place for other groups showed no enhancement in their Memory index when they experienced this moving around one hour after the reading (Fig. 2A). Similarly, there was no memory improvement in the groups of students which had been previously informed of the contents of the novel class (Fig. 2B).

**Figure 3:**
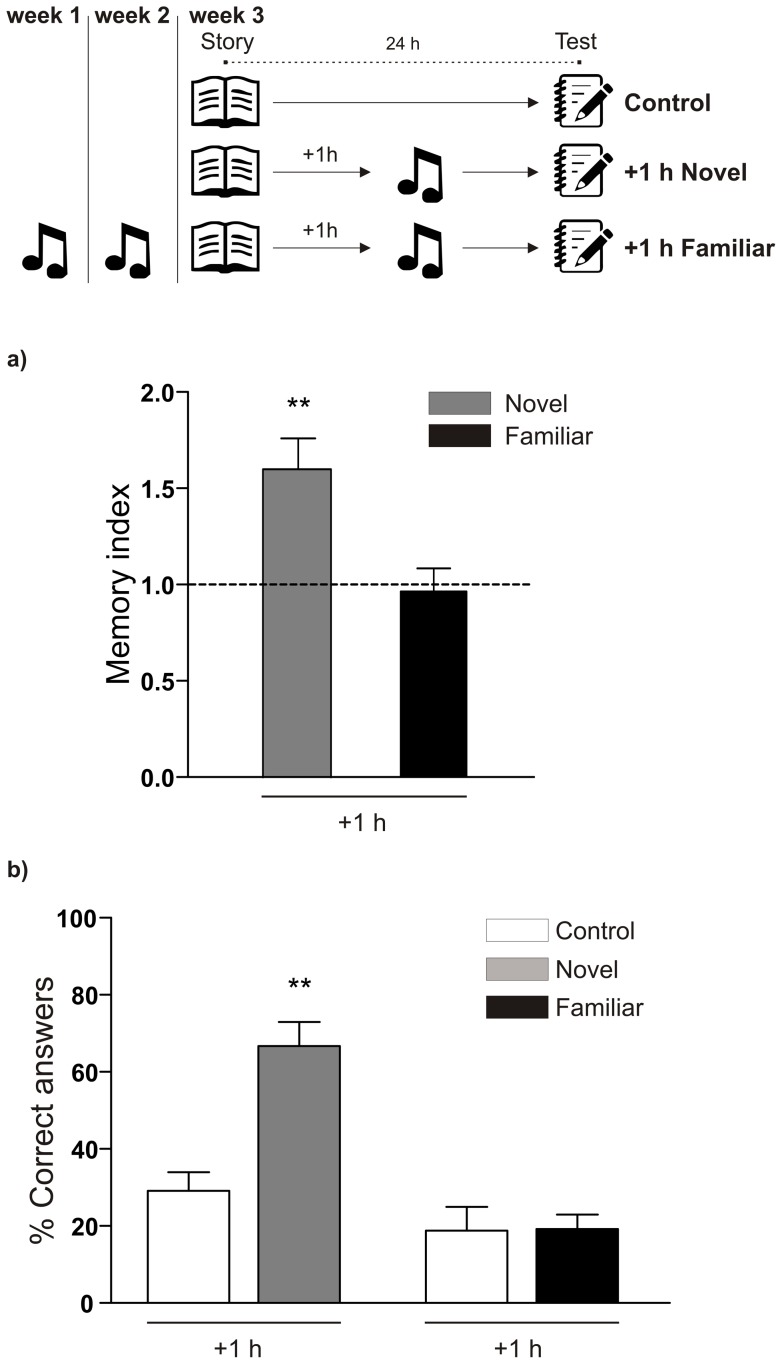
Memory of the story is enhanced by a novel, but not a familiar, music lesson. Schematic representation of the experimental protocol is presented on the top of the panels. A) Memory index for the story is shown as mean ± SEM of the ratio between data from groups of students that experienced novel (n = 33) or familiar music lesson (n = 40) and control students (dotted line, n = 52). A short story was read and a novel music lesson or a familiar music lesson was given 1 hour later. LTM of the story was evaluated 24 hours after. ** p<0.01 vs Control and Familiar group, Newman-Keuls analysis after one-way ANOVA. B) Correct answer percentages corresponding to hard level of difficulty questions calculated from students analyzed in A (n = 6 for all groups). ** p<0.01 vs Control, Student's t test.

We then wondered to know if another type of novelty, in the form of a music lesson, given 1 hour after the reading of the story, could also enhance memory consolidation. As expected, this kind of novelty also improved the narrative memory (Fig. 3A). Interestingly, the enhancing effect was not observed when the lesson was familiar (Fig. 3A). For this purpose, the music lesson had been repeatedly given for two weeks. In the third week the story was read to the students one hour before the familiar music lesson. The percentage of correct answers on the hard questions was significantly increased for the students in the novelty group; however, if the students were familiarized to the music lesson, such effect was not observed (Fig. 3B). Intermediate difficulty and easy questions were answered by a similar proportion of students in both groups (Fig. S3). These results show that the enhancing effect is specifically related to the novel nature of the music lesson and not to the lesson itself.

**Figure 4:**
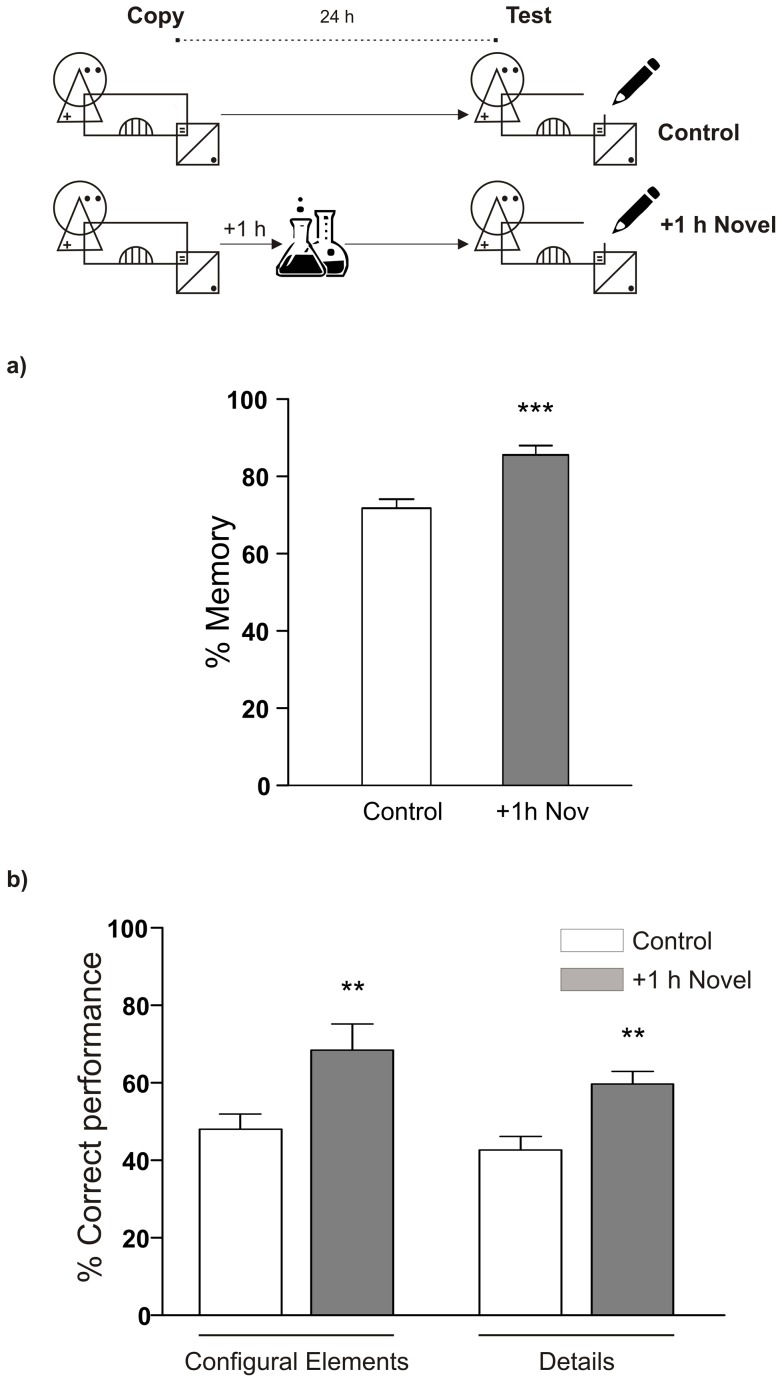
A graphical memory can also be enhanced by the experience of a novel science lesson. Schematic representation of the experimental protocol is presented on the top of the panels. A) LTM for the complex figure is shown as mean ± SEM of memory percentage between the copy and the recall of the figure at the test. Comparisons were made between control (n = 32) and novel groups (n = 43). *** p<0.001 vs Control, Student's t test. B) Mean ± SEM of the correct performance percentages corresponding to configural elements and details of the complex figure are shown for control and novel groups analyzed in A. ** p<0.01 vs Control, Student's t test.

Finally, to analyze the generality of the phenomena, we selected a different type of learning based on graphical information. Therefore, we implemented an adaptation of the Rey-Osterrieth's complex figure task for children [17]. The procedure followed was the same as before. Students were allowed to copy a figure for 2 minutes and 24 hours later they were asked to draw it again in 4 minutes, based on what they remembered of it. The individual retention score was calculated for each item in the figure drawn in the test session by comparing them with the figure drawn the day before (See Text S1). Analysis of the drawings showed a significant enhancement in the retention for those students who experienced a novel science lesson one hour after copying the figure (Fig. 4A). Interestingly, the improvement was observed in the retention of the Rey-Osterrieth's complex figure as a whole, since the number of correct items was increased in both configural objects and in details as well (Fig. 4B).

## Discussion

Different strategies have been used in an attempt to improve cognitive functions, (e.g. executive functions, memory, attention, processing speed) through transference, generalization and retrieval [19], [20], [21], [22], [23]. In that sense, numerous games (e.g. Brain Age, Big Brain Academy, and Brain Challenge), web pages and computer programs (e.g. Luminosity, Happy neuron, Who has the biggest brain?, Brain training, etc) attract thousands of users under the promise of an accelerated enhancement in several cognitive functions (increased working memory, increased fluid intelligence, etc), encouraging them to undergo intensive training [24]. Even in the case of positive outcomes, there is not such a project of these characteristics in the educative field. Here we show a consistent enhancement of memory in students of different ages using a quick non-expensive methodology (it only requires a novel session of 20 minutes) that can be easily implemented by teachers in the school setting.

Our results show that educationally relevant novel events experienced during normal school hours can improve LTM for tasks learned during regular school lessons. This effect is restricted to a critical time window around learning and is particularly dependent on the novel nature of the associated experience. In addition, we show that this memory enhancement procedure is applicable to at least two different types of memory, one narrative (verbal) and the other graphical (visual), by different kinds of novel experiences. Furthermore, these findings suggest that memory improvement induced by novelty is not derived from an increase in the arousal state or from lowering the threshold to learn, because both types of novel experiences can exert their positive effect on memory even when they occur after the acquisition of the information to be remembered.

There is a well accepted idea establishing that the entrance of new information into LTM depends on neural signals triggered by the novel or rewarding aspects of the stimulus to be encoded [25], [26]. Our findings are in consistent with these observations and show that the role of novelty is broader than it was previously thought. In all, we demonstrate that novel lessons exert a penumbra-like effect on the surrounding events that can improve the LTM of students for information acquired in temporal proximity of novelty. Based on “synaptic tagging and capture” hypothesis [27], [28], [29], [30], [31], we have shown in rodents that novel events late-associated to a weak learning synthesized proteins which could be captured by the learning tags set by the weak training, resulting in the promotion of LTM formation for this event [12], [14], [16]. This phenomenon was named “behavioral tagging” and it was demonstrated the existence of a window of sensitivity to novelty which could be explained in terms of the transient nature of the learning tag induced by the weak training and also by the kinetics of production and degradation of plasticity-related protein induced by novelty. Here, we show that novel lessons can exert late-associative effects on LTM formation for the story telling or figure drawing, leading to the consolidation of their memories. Though it cannot be demonstrated in this work that the effect of novelty depends on protein synthesis, we put forward the hypothesis that the learning experience (story/drawing) triggers a transient process (which resembles the idea of a learning tag) that enables the consolidation of those information by the effects of the novel experience occurring around a critical time window. Further studies are required on the identity of the tag and its location. Our results show that novelty has a symmetrical effect on the promotion of LTM formation, whether it occurs 1 h before or after the acquisition of the information, but it does not exert any effect on LTM promotion if it is experienced at distant time points of the acquisition. Moreover, when the two stories were read to the same groups of students, novelty *specifically* exerted a promoting effect on the memory of the story read 1 h before and not on the other story that had been read 4 h before. These results suggest that when these events (story and novelty) are temporally more distant, the processes triggered by both of them probably do not interact. Besides, the promoting effect of novelty on memory formation depends on the novel features of the lesson; when it is familiar, it lacks efficacy. Therefore, taken as a whole, our findings suggest that a behavioral tagging-like process could be operating in humans. Alternatively, any other brain mechanisms based on factors triggered by a novel lesson acting on an anterograde and retrograde critical time window close to the learning session could explain the promoting effects on its LTM formation.

Finding different teaching strategies to improve students' performance is a subject of great relevance to education. Our findings provide a tool that could be easily transferred to the classroom by the incorporation of educationally novel events in the school schedule as an extrinsic adjuvant of other information acquired some time before or after it. This approach could be a helpful tool for the consolidation of certain types of topics that generally demand a great effort from the children.

## Supporting Information

Figure S1 **The percentage of intermediate difficulty questions correctly answered is increased when students experience a novel science lesson around the time of the reading.** Schematic representation of the experimental protocol is presented on the top panels. A novel Science lesson was given at different times before (−4 h, −1 h) or after (+1 h, +4 h) the reading of a short story. 24 hours later it was evaluated how much the students remembered on the story by means of a written test. Means ± SEM of the percentage of correct answers to easy (−4 h, n = 8; −1 h, n = 12; +1 h, n = 12; +4 h, n = 12) and intermediate (−4 h, n = 6; −1 h, n = 9; +1 h, n = 9; +4 h, n = 9) levels of difficulty questions are shown corresponding to the same groups of students analyzed in Figures 1A and B. Comparisons were made between control and novel groups for each of the time points. * p<0.05, ** p<0.01 vs Control, Student's t test.
(TIF)

Figure S2 **The percentage of correct answers is increased when students experience a novel science lesson in a specific time lapse around the task.** Schematic representation of the experimental protocol is presented on the top of the panels. Figures depict the mean ± SEM of the percentage of correct answers to easy (+4 h, n = 8; +1 h, n = 8) and intermediate (+4 h, n = 6; +1 h, n = 6) levels difficulty questions from the same groups of students analyzed in Fig. 1C and D. Students experienced a novel science lesson in different times (+1 h and +4 h) from a particular story (A or B) telling,. Half of the students were tested on the story A and the rest on the story B. * p<0.05 vs +4 h.
(TIF)

Figure S3 **Percentage of correct answer is not enhanced when the concurrent lesson is familiar.** Schematic representation of the experimental protocol is presented on the top of the panels. Memory for the story is shown as mean ± SEM of the ratio between data from groups of students that experienced novel or familiar music lesson and control students. A short story was read and a novel music lesson or familiar music lesson was given 1 hour later. 24 hours later it was evaluated how much the students remembered about the short story. Correct answer percentages corresponding to easy (n = 8 for all groups) and intermediate (n = 6 for all groups) levels of difficulty questions are shown for the same groups of students analyzed in Fig. 2 compared against its control group. p>0.05 vs. Control, Student's t test.
(TIF)

Picture S1 **Rey- Osterrieth's complex figure for children.** Students were given this image to copy during acquisition session and 24 hours later their memory for the figure was tested by asking them to draw what they recalled of it (test session). In this figure we considered 4 Configural elements, here pictured in black (circle, triangle, rectangle and square), and the 7 detail elements, here pictured in green (two dots, a cross, semicircle, 4 lines inside the semicircle, diagonal inside the square, black dot inside the square and equal symbol).
(DOC)

Table S1 **Characteristics of the institutions and the students intervening in the experiments.** Detailed information on the participating school shifts, age and number of the students. All the students attend to only one shift in these schools. The schools that offer both morning and afternoon shifts, have separate groups of students in the respective morning and afternoon shifts.
(DOC)

Table S2 **Scoring criteria for Rey-Osterrieth Figure Test drawing.** Here we present the general scoring criteria for drawing the ROCF(*2*). If the student completes all the figures and places them in the right position, the corresponding score will be of 22 points.
(DOC)

Text S1 **Supplementary methods.**
(DOC)
