# Supplementary figures and images for: Memory in Elementary School Children Is Improved by an Unrelated Novel Experience
Source: PLoS One. 2013 Jun 19;8(6):e66875. doi: 10.1371/journal.pone.0066875 (PMC3686730; doi:10.1371/journal.pone.0066875)

**Picture S1**


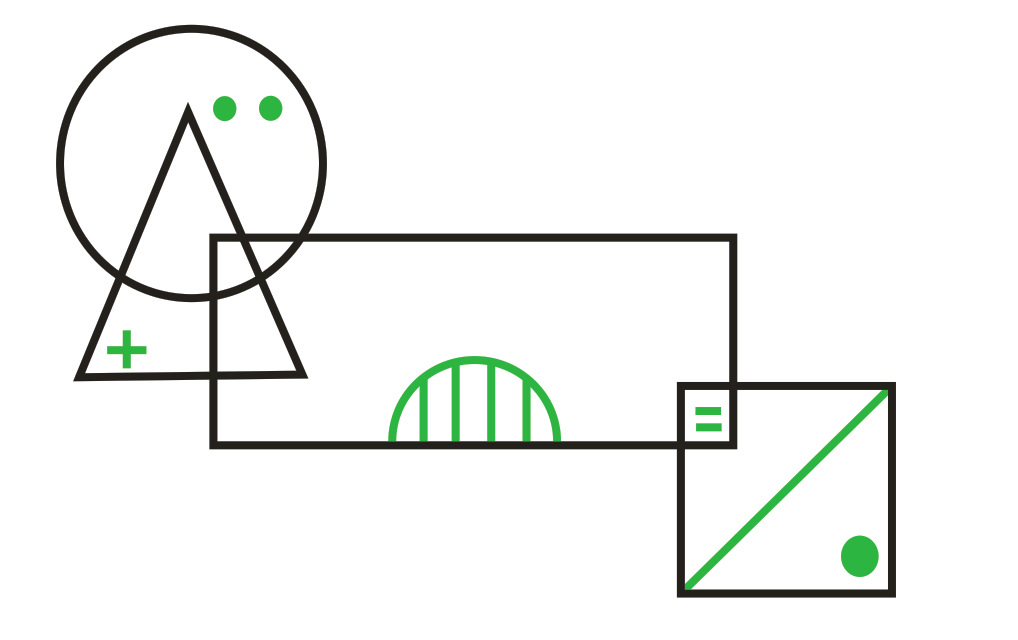

Supplement: Picture S1 — Rey- Osterrieth's complex figure for children. Students were given this image to copy during acquisition session and 24 hours later their memory for the figure was tested by asking them to draw what they recalled of it (test session). In this figure we considered 4 Configural elements, here pictured in black (circle, triangle, rectangle and square), and the 7 detail elements, here pictured in green (two dots, a cross, semicircle, 4 lines inside the semicircle, diagonal inside the square, black dot inside the square and equal symbol). (DOC) [file pone.0066875.s004.doc]
