# Supplementary material for: Memory in Elementary School Children Is Improved by an Unrelated Novel Experience
Source: PLoS One. 2013 Jun 19;8(6):e66875. doi: 10.1371/journal.pone.0066875 (PMC3686730; doi:10.1371/journal.pone.0066875)
Supplement: Table S1 — Characteristics of the institutions and the students intervening in the experiments. Detailed information on the participating school shifts, age and number of the students. All the students attend to only one shift in these schools. The schools that offer both morning and afternoon shifts, have separate groups of students in the respective morning and afternoon shifts. (DOC) [file pone.0066875.s005.doc]

**Table S1.**

| **Institution** | **Year of the activities** | **School shifts** | **State/**  **Private** | **Religious** | **Bilingual** | **Age of the students (years)** | **Number of students** |
| --- | --- | --- | --- | --- | --- | --- | --- |
| 1 | 2009 to  2010 | Morning- Afternoon | State | No | No | 8 | 142 |
| 2 | 2009 to 2010 | Morning- Afternoon | Private | No | No | 7 and 8 | 199 |
| 3 | 2010 | Morning- Afternoon | Private | Yes | No | 8 and 9 | 347 |
| 4 | 2010 | Morning- Afternoon | Private | No | Yes | 7 and 8 | 247 |
| 5 | 2009 to 2011 | Afternoon | Private | Yes | No | 7,8 and 9 | 307 |
| 6 | 2010 | Morning- Afternoon | Private | No | No | 7,8 and 9 | 296 |
| 7 | 2010 | Afternoon | Private | No | Yes | 9 | 103 |
| 8 | 2011 | Morning | Private | No | Yes | 7 | 35 |
