# Supplementary material for: Memory in Elementary School Children Is Improved by an Unrelated Novel Experience
Source: PLoS One. 2013 Jun 19;8(6):e66875. doi: 10.1371/journal.pone.0066875 (PMC3686730; doi:10.1371/journal.pone.0066875)
Supplement: Table S2 — Scoring criteria for Rey-Osterrieth Figure Test drawing. Here we present the general scoring criteria for drawing the ROCF(2). If the student completes all the figures and places them in the right position, the corresponding score will be of 22 points. (DOC) [file pone.0066875.s006.doc]

**Table S2.**

| **Score** | **Accuracy** | **Placement** |
| --- | --- | --- |
| 2 | Accurately drawn | Correctly placed |
| 1 | Accurately drawn | Incorrectly placed |
| 1 | Inaccurately drawn | Correctly placed |
| 0.5 | Inaccurately drawn, but recognizable | Incorrectly placed |
| 0 | Inaccurately drawn and unrecognizable, or omitted | Incorrectly placed |
