# Supplementary material for: Memory in Elementary School Children Is Improved by an Unrelated Novel Experience
Source: PLoS One. 2013 Jun 19;8(6):e66875. doi: 10.1371/journal.pone.0066875 (PMC3686730; doi:10.1371/journal.pone.0066875)
Supplement: Text S1 — Supplementary methods. (DOC) [file pone.0066875.s007.doc]

**Supplementary Methods**

**Students, institutions and general procedures**

1676 students (aged between 7-9 years old) from 8 different schools in Buenos Aires, Argentina were evaluated. 786 students participated in the activities belonging to Fig. 1, 102 students in the activities belonging to Fig. 2, 171 students in the activities belonging to Fig. 3 and 198 in the activities belonging to Fig. 4. The remaining 25% of the total students participated in preliminary experiments aimed to optimize the procedures and was not included in the data reported in the present work. Characteristics of the institutions and the students intervening in the experiments are shown in Table S1. Procedures were reviewed and approved by the academic authorities of each participating educational institution.

Literary and graphical activities were performed by each group’s own teacher in their corresponding classrooms as part of a regular class. Teachers were not informed of the aims of the research nor of the results. They were only given a written instructive with the basic information to carry out each task. In every case there has been an intensive follow-up of the activities in the school by the researchers. Both types of novel lessons –science and music- were in charge of the researchers and of a music therapist respectively. These activities took place outside the classroom, in an area not habitually frequented by students. Tests were corrected by researchers blind to the experimental condition of the students. Students have not been informed before a hand about the procedures to avoid any specific encoding strategy and to minimize stress.

Each school has a fixed syllabus’ schedule established from the beginning of the year. Every school day is divided in three classwork modules with two breaks of 15 min. each between them, both in the morning and in the afternoon shifts. Contents from the modules belong to the following areas: “language, maths, social sciences, life sciences, english (as a foreign language), artistic formation and physical education”. Each course has its own schedule, which may differ between institutions. Literary or graphic activities (which last for 5 min.) were performed in a random fashion in any of those modules.

**Description of the experimental procedure in Fig. 1A: Temporal characterization of the effect of novelty on the literary memory.**

A literary memory study was carried out**.** A short story was read by the teacher to her group students during their normal daily activities in the classroom (Control). The reading took place only once and was made following the same outline as their habitual classwork. In parallel, another group of students belonging to the same Institution and of the same grade was read another short story, which was associated to a novel lesson (Novelty). Twenty-four hours later and without any previous notice (neither at the reading nor at the test), long-term memory (LTM) was evaluated in both groups using a written list of 10 questions of different difficulty levels -easy, intermediate and hard- related to the story read the day before. Just like the reading, the test took place at each course own classroom. Students were not informed of the test to prevent exerting a higher arousal state during the reading or motivating any type of conscious effort or particular method to enhance the memory of the story with the aim of getting a good score. Besides, students were asked to answer the questionnaires individually as an activity that did not carry any marks so that it did not represent a stressful event for them.

The novel activity was given at different time points before or after the reading of the story (-4 hr, -1hr, +1hr, +4hr). As an example, here we show the experimental procedure for one of the Schools. If group 1 was read the story A, then group 2 was simultaneously read the story B (each group being at their usual classroom) but afterwards, this group 2 was taken to a novel place to experience the science class. Two weeks after this first stage, stories and treatments were interchanged between groups. In consequence, group 1 would experience the novel class and be read story B in the second stage of the experiment. Likewise, group 2 was read story A but did not experience novelty. Thus, activities were designed and performed in a balanced way, being each of the courses subject to both conditions (Control and Novelty) with a 14 day lapse between them. Data was included in the analysis if the scores obtained in the control conditions were similar between both groups (this was observed in 95% of the cases and allows us to conclude that both stories had a comparable level of complexity and obtained similar correct answer percentages in control conditions).

There were 330 participants in the novel groups and 322 participants in the control groups. As it is explained above, each time point of the curve had its specific control whose results are expressed as (mean ± SEM): -4 h Control group (n=56; 1,002 ± 0,1034); -1 h Control group (n=55; 1,001 ± 0,1009); + 1 h Control group (n=113; 0,999 ± 0,0606); +4 h Control group (n=98; 1,001 ± 0,0544). With the aim of comparing the effect of novelty in time we performed a one-way ANOVA, randomly selecting a group of 89 control students (1,013 ± 0,0890 ) to avoid disparities with the size of the experimental groups.

**Description of the experimental procedure in Fig. 1C: Specific time-task protocol.**

Using a similar approach as described before, students were read two stories (A and B) on the same day, with an interval of three hours between sessions. An hour after the second short story had been read, a novel science class was presented. Twenty-four hours later, LTM for one of the stories was evaluated on one half of the students while the other story was evaluated on the second half. As a control, parallel groups of students were read two different stories (randomized alternate) separated by a 3 hours interval. Twenty-four hour later, LTM was tested for both stories as described above.

In this activity there were 134 participants, being 48 of them represented in Fig. 1C and 1D and the remaining 86 participated in the control condition without novelty (as reported in the main text).

**Description of the experimental procedure in Fig. 2A: Pre-informed protocol.**

To reduce the impact of the novel experience, students were informed of the contents of the novel science lesson just after the reading of the story. Just as done with other groups, one hour after the reading they were taken outside their classroom to experience the novel class. The LTM for the story in this group was similar to the control group that did not experience any novelty.

There were 22 participants in the pre-informed group and 25 participants in the control group.

**Description of the experimental procedure in Fig. 2B: Moving around protocol.**

Using a similar approach as described before, the teacher read the story and 1 hour later, he/she conducted the students to the environment where the novel class would take place, but without experiencing it. Immediately after their arrival to this place, they returned to the classroom. This group was tested the following day and the results were comparable to control students that did not move around their classroom. In this activity there were 30 participants in the moving group, 25 in the control group.

**Description of the experimental procedure in Fig. 3: Effect of a novel or familiar music lessons on literary memory formation.**

A group of students was read by their teacher one of the stories which could (Novelty) or could not (Control) be in association with a novel music lesson was given 1 hour later. Twenty-four hours later, LTM was evaluated in both groups using written list of 10 questions related to the story read the day before, as mentioned above. Just as performed in Fig. 1A, both experimental procedures and stories were interchanged between groups. The same method was followed with the familiar music lesson, given one hour after the reading of the story (Familiar Group).

In this activity there were 33 participants in the novel group, 40 in the familiar group and 98 control students. Each treatment had its specific control expressed as (mean ± SEM): Novel Control Group (n=53; 1,002 ± 0,0832) and Familiar Control Group (n=45; 1,003 ± 0,1236). With the aim of comparing the controls with novelty and familiarity effects we performed a one-way ANOVA, randomly selecting a group of 52 control students (1,002 ± 0,0925) to avoid disparities with the size of the experimental groups.

**Description of the experimental procedure in Fig. 4: Effect of novelty on a graphical memory.**

We designed a specific task to test graphic memory based on Rey-Osterrieth's complex figure test (ROCF) [1]. The teacher gave each student a printed sheet with the complex figure picture (drawn all in black) on the top part of it (Picture S1). Each student was allowed for a two-minute period to copy the figure on the bottom of the sheet. Once finished, drawings were handed in to the teacher (Control). Twenty-four hours later, the same teacher gave each student a blank sheet of paper to test the memory of this figure by asking students to draw it again in a 4-minute interval. In parallel, a group of students of the same level and school followed the procedure but one hour after having copied the figure they experienced a novel science class (+1h Nov). Twenty-four hours after memory of the figure was tested in this Novel group with the same procedure followed for the Control group.

We considered only the tests of those students who had correctly reproduced 75% of the Complex Rey’s Figure in the copy session. To assess the effect of novelty on the graphic memory formation both in configural objects and details as well. For this reason, only 75 students out of the 198 participants were included in the test.

**Stories**

Two different stories adapted from textbooks commonly read in primary school were used in this work. Both share similar narrative characteristics such as main and secondary characters, complexity of the plot and details (scenery, particular information on the characters, etc.) and are aimed to the same audience. Both stories were written in Spanish and activities were dictated in Spanish as well, being it the mother tongue of the students. Here we present a brief outline translated to English of each of them:

**Story A**

Boris worked as a vampire but his duty was not easy provided that he lived in a city whose habitants went early to bed. So food was always scarce. He lived in a dark castle and woke up every midnight to go out in search of a victim. One particular night, he saw an apparently innocent old lady and went right over her. He had his mouth wide open when the lady started to hit him with her large and tough hands. Unbelievable! Boris was being defeated by the local dentist, who was famous for her ability to treat even the worst cases. She grabbed him by the neck and took him to her dental clinic. Shortly after, he was sitting on the chair under the vigilant look of the dentist. She fixed all the cavities he had and filed his fangs until they were almost normal. Boris had a whole new look with his shiny smile. He decided to quit his job as a vampire and since then, his teeth are always white as milk.

**Story B**

The story is about a character named Gerome who is the bulletman of a local circus. The act he usually performs is to be fired high with a large cannon. One day the cannon had been overloaded with gun powder and Gerome was thrown really high. He fell on a distant farm and was warmly welcomed by the farm owners, who offered him a place to stay until the circus passed by that village. Gerome felt at ease in the farm where fresh vegetables were grown -such as tomatoes, corn and carrots- and he decided to stay. He quickly learned all the farm chores and became a great helping hand. He was delighted with the homemade food. Three years later, the circus arrived in the town. Gerome went back to the circus crew. He couldn’t perform his act because he got stuck by his greatly enlarged belly, a consequence of his recently developed love for food. After this shameful success Gerome took up a new career as a baker and was famous for his specialty: the cannoli.

**Assessment**

Memory for the literary activity

To test the LTM for the stories, students were given a written list of 10 questions of different difficulty levels - 4 easy, 3 intermediate and 3 hard- related to the story read the day before. Questions were specifically designed by the researchers to fit with this levels’ classification. We consider that the percentage of correct answers obtained in control groups serves as a confirmation that levels were of different difficulty. Easy questions were correctly answered by 80% of the students in the control groups, intermediate difficulty questions were correctly answered by about 40% of the students in the control groups and hard difficulty questions were correctly answered only by 20% of the students in the control groups (Figures S1-3).

**Easy questions** were taken as a positive control of the students’ attendance to the lesson and their comprehension of the story. They were based on central aspects of the story related to the main character (name, occupation, clothing, etc.) and core events in the plot. For example: “Where did Boris live?” (Story A) or “What did Gerome work as?” (Story B).

**Intermediate difficulty questions** addressed relevant aspects of the story, although only mentioned once, or other aspects mainly related to secondary characters or to the place where the story took place. For example: “What time did Boris wake up?” (Story A) or “What types of tasks did Gerome learn at the farm?” (Story B).

**Hard difficulty questions** were made on details and events unrelated to the core of the plot that would commonly/ normally be forgotten. For example: “What was exactly the treatment the dentist gave to Boris?” (Story A) or “Which vegetables were grown at the farm where Gerome fell?” (Story B).

Questionnaires were corrected by the researchers –not the teachers- who were blind to the treatment of the students. One point per each correct answer was assigned, and **Memory** **index** was calculated as the ratio between the score obtained for both the hard and the intermediate questions for students belonging to the Novelty group relative to the mean obtained for its corresponding Control group. Both short stories were appropriate for the instruction level of the students and had a comparable complexity (this assumption was based on the number of correct answers from Control groups).

The other parameter analyzed in this study is **the percentage of correct answers** classified by levels of difficulty. We considered three questions of each level, excluding from this analysis one of the easy questions in order to make comparisons of this level with the others. This was calculated as follows: first, it was obtained the proportion of correct answers for each separate category –easy, intermediate and hard- for each group of students. Afterwards, a mean of the proportions from all the courses was obtained for each category. These means were used to compare the scores between Control and Novelty groups (i.e. to analyze the effect of novelty in each category) in the intermediate and hard questions. To illustrate the procedure, here we include an example: to calculate the number of correct answers from a group of 30 students in control or novelty conditions, there were 90 possible answers (three questions per each student, corresponding to one level of difficulty) in each condition. If control group correctly answered 18 questions out of 90, then their score is 20%. If the novelty group correctly answered 54 out of that 90 questions, their score is 60%.

**Memory for the Complex figure**

Each of the elements in the figure was analyzed for location, accuracy and organization by using the Rey-Osterrieth's score [2] and an individual score for each student was obtained following the criteria in Table S2, being 22 points the maximal score. Only those students who had faithfully copied more than 75% of the drawing were included in the experiment. **Memory percentage** for each student was calculated as the proportion between the score obtained for the copy and the drawing in the test session. Another parameter analyzed in this experiment is the **correct performance percentage** classified by levels of visual complexity. **Configural elements** are considered to be the most “global” parts of the design (See Picture S1, black geometrical figures), the **details** as the most “local” elements (See Picture S1, green figures). An average was obtained from the individual performances for both configural elements and for details.

**Novel lessons:** A teacher unknown to the students taught them a novel lesson of 20 minutes without previous notice and in a context which markedly differed from their usual classroom but still inside the school building. Two kinds of novel classes were given, one of them being a science lesson and the other a music lesson. Both types of lessons had been specially designed to have comparable characteristics regarding students´ participation and novelty of the stimuli. These novel lessons were given at different time points before or after the reading of the story (1 or 4 hours).

In this work we considered it to be a novel activity if the class/lesson (science or music) complied with these requirements:

1. The whole group of students was unexpectedly taken from its classroom and led to a different place to attend a lesson that was not previously informed about until it started.
2. This novel lesson was given in a place inside the school but not usually frequented by the students for their lessons –e.g. multiple uses room, laboratory, patio-.
3. The lesson was given by a skilled teacher unknown to the students.
4. It is a short activity (20min) never experienced before by the students, with novel contents appropriate for their age.
5. Students are encouraged to actively participate and be attentive at all times. When the activity finishes, they return to their habitual classroom.

The novel science class was based on simple experiments aiming to the constant participation of the students and their full interaction with the elements. In the first years at school it is extremely infrequent to experience this kind of empirical science lessons. In the class, students were introduced to some basic physics and biology principles (such as density, gravity, superficial tension and electrostatics) with playful activities. The novel science lesson was specially designed and given by the researchers. Besides, the novel music class was planned and dictated by the music therapist aiming to resemble those novel features of the science class. Likewise, students were not informed of this class until its start and were involved in its activities. Considering that students habitually have music lessons in their schools (as it is a compulsory subject), the novel music lesson was based on innovative teaching methods and it also dealt with topics highly infrequent for the students. The lesson consisted of games with homemade instruments (like rods, x-ray films, buckets and balls) and it was intended to show that music could even be made with simple elements and yet they could also form an orchestra with those atypical instruments.

**Familiar lesson**

In this work we define a familiar lesson as a lesson that had been repeatedly given and whose contents and teachers were already known to students**.** The same music lesson described above was presented for 2 weeks (one lesson each week) and in the third week this "familiar lesson" was associated with a short story read 1 hour before the music lesson.

**References for Supplementary Methods**

1. Rey A (1959) Test de copie et de reproduction de memoire de figures geometriques complexes [A test of copy and recall of a complex geometric figure]. Editions du centre de Psycholegie Appliquee.

2. Shin MS, Park SY, Park SR, Seol SH, Kwon JS (2006) Clinical and empirical applications of the Rey-Osterrieth Complex Figure Test. Nature Protocols 1, 892-9.
